# Supplementary material for: Reconciling Mining with the Conservation of Cave Biodiversity: A Quantitative Baseline to Help Establish Conservation Priorities
Source: PLoS One. 2016 Dec 20;11(12):e0168348. doi: 10.1371/journal.pone.0168348 (PMC5173368; doi:10.1371/journal.pone.0168348)
Supplement: S1 Dataset — (ZIP) [file pone.0168348.s002.zip › Taxa/Serra Sul/SS_2010/S11D_65.pdf]

S11D-65		1ª	AB	2ª	AB	ZON
Arthropoda						
Arachnida						
Acari						
Parasitiformes						
Mesostigmata						
Laelapidae	sp.3	1				E
Mesostigmata	sp.4	1				E
Opilioacarida						
Opilioacaridae	sp.1	1				E
Amblypygi						
Phrynidae	jovens	1	0,1429			E
Heterophrynus	sp.	1	0,1429			
Araneae						
Araneidae	jovens	2				E
Alpaida	*septemmammata*			1		E
Ctenidae	jovens			2	0,2222	E
Ochyroceratidae	jovens	2				E
Oonopidae						
gr. *Xycarphius*	sp.3			1		E
Pholcidae						
aff. *Ibityporanga*	sp.1	1		1		E
Ninetinae	sp.1			1		E
Salticidae	jovens	1				E
Amphidraus	sp.1	1				E
Tetrablemmidae						
Matta	sp.1			1		E
Theridiosomatidae	jovens	1				E
Plato	sp.1	1				E
Opiliones						
Laniatores						
Stygnidae	jovens	1	0,1429			E
	sp.1			1	0,1111	E
Pseudoscorpiones						
Chernetidae						
Spelaeochernes	sp.1	1				E
Diplopoda						
Polydesmida						
Paradoxosomatidae	sp.2	1	0,1429			E
Entognatha						
Diplura						
Campodeidae	sp.1			1		E
Insecta						
Coleoptera	jovens			1		E
Staphylinidae						
Pselaphinae	sp.1	1				E
Collembola						
Arthropleona						
Entomobryoidea						
Paronellidae	sp.1	1				E
Diptera						
Nematocera	jovens	1				E
Cecidomyiidae						
Cecidomyiinae	sp.	1				E
Mycetophilidae						
Orfelia	sp.	1				E
Psychodidae						
Sciopemyia	*sordellii*			1		E
Hemiptera						
Heteroptera						
aff. Pyrrhocoroidea						
Reduviidae	jovens	1	0,1429	2	0,2222	E
Homoptera						

Cixiidae	sp.3			1		E
Hymenoptera						
Vespoidea						
Formicidae						
Apterostigma	sp.1	1				E
Camponotus	sp.1	2				E
Gnamptogenys	*striatula*			1		E
Pheidole	sp.2	2		1		E
Lepidoptera	jovens	1				E
Neuroptera						
Mantispidae	sp.	1	0,1429			E
Orthoptera						
Ensifera						
Paraclodes	sp.1			4	0,4444	E
Psocoptera						
Psocomorpha	jovens	1				E
Thysanura						
Ateluridae	jovens			1		E
	sp.1	1				E
Nicoletiidae	jovens	1				E
Chordata						
Amphibia						
Anura						
Neobatrachia						
Strabomantidae						
Pristimantis	*fenestratus*	1	0,1429			
